# Short-term effects of ambient air pollution on emergency department visits for urolithiasis: A time-series study in Wuhan, China

**DOI:** 10.3389/fpubh.2023.1091672

**Published:** 2023-01-30

**Authors:** Haoyue Xu, Yaqi Liu, Jianing Wang, Xiaoqing Jin

**Affiliations:** ^1^The Emergency Center, Zhongnan Hospital of Wuhan University, Wuhan, Hubei, China; ^2^The Second Clinical School, Wuhan University, Wuhan, Hubei, China

**Keywords:** air pollution, urolithiasis, emergency department visits, time-series study, public health

## Abstract

**Background:**

Previous studies have explored the correlation between short-term exposure to air pollution and urinary system diseases, but lack of evidence on the correlation between air pollution and urolithiasis.

**Methods:**

Daily data of emergency department visits (EDVs), concentrations of six air pollutants (SO_2_, NO_2_, PM_2.5_, PM_10_, CO, and O_3_) and meteorological variables were collected in Wuhan, China, from 2016 to 2018. And a time-series study was conducted to investigate short-term effects of air pollutants on urolithiasis EDVs. In addition, stratified analyses by season, age and gender were also conducted.

**Results:**

A total of 7,483 urolithiasis EDVs were included during the study period. A 10-μg/m^3^ increase of SO_2_, NO_2_, PM_2.5_, CO, PM_10_, and O_3_ corresponded to 15.02% (95% confidence interval [CI]: 1.69%, 30.11%), 1.96% (95% CI: 0.19%, 3.76%), 1.09% (95% CI:−0.24%, 2.43%), 0.14% (95% CI: 0.02%, 0.26%), 0.72% (95% CI: 0.02%, 1.43%), and 1.17% (95% CI: 0.40%, 1.94%) increases in daily urolithiasis EDVs. Significant positive correlations were observed between SO_2_, NO_2_, CO, and O_3_ and urolithiasis EDVs. The correlations were mainly among females (especially PM_2.5_ and CO) and younger people (especially SO_2_, NO_2_, and PM_10_) but the effect of CO was more obvious in elders. Furthermore, the effects of SO_2_ and CO were stronger in warm seasons, while the effects of NO_2_ were stronger in cool seasons.

**Conclusion:**

Our time-series study indicates that short-term exposure to air pollution (especially SO_2_, NO_2_, CO, and O_3_) was positively correlated with EDVs for urolithiasis in Wuhan, China, and the effects varied by season, age and gender.

## 1. Introduction

Urolithiasis, a common urinary system disease, including stone in kidney, ureter, bladder, and urethra. Although rarely life-threatening, urolithiasis can often cause intense pain and is associated with increased risks of urinary tract infection, hydronephrosis, and renal function decline (1). In recent decades, urolithiasis's incidence rate and prevalence have continued to rise worldwide and its burden mainly concentrated in Asia and Eastern Europe (41). In the analysis of geographical differences, the prevalence rates of kidney stones were higher in the hot south and southwest regions of China (3). A present study indicated that currently kidney stones affect about one in 17 adults (the adjusted prevalence rate was 5.8%; 6.5% in men and 5.1% in women) in China (3). Renal colic caused by urolithiasis is a common complaint in the emergency department and a significant burden for healthcare systems and emergency departments (4), and it is estimated that this burden is likely to increase over time (39). Therefore, an improved understanding of the risk factors of urolithiasis is of great significance to public health. Some studies have explored the correlation between urolithiasis and diet habits, obesity, diabetes, metabolic syndrome, fluid intake, and environmental factors, such as geographical and meteorological factors (5–7). Among them, the correlations between seasonal changes, ambient temperature and urolithiasis have been extensively studied (7). However, the correlation between air pollutants, the important components of the exposed air environment, and urolithiasis has rarely been explored.

In recent years, air pollution has been considered as one of the crucial factors threatening human health. Exposure to ambient air pollution is associated with an increased risk of global mortality and incidence rate from various diseases, especially in developing countries (8, 9). Several studies have also reported that air pollutants may affect renal function through oxidative stress, inflammatory responses, and other mechanisms, leading to the development of a range of urological diseases, such as chronic kidney disease and renal failure (10, 11, 40). Oxidative stress and inflammation have been suggested as the potential mechanistic pathways affecting stone formation (12, 13). Therefore, we hypothesized that exposure to air pollution might also be correlated with an increased risk of urolithiasis. Our study will be beneficial for hospitals to understand the potential risk of urolithiasis from exposure to ambient air pollution to take precautions and maintain order on particularly heavily polluted days.

Given the above contents, we conducted a time-series study on the correlations between six ambient air pollutants (SO_2_, NO_2_, PM_2.5_, PM_10_, CO, and O_3_) and emergency department visits (EDVs) for urolithiasis in Zhongnan Hospital of Wuhan University from January 1, 2016 to December 31, 2018. Season, gender and age differences were considered to examine the adverse health effects of air pollution on different groups. Furthermore, we also drew exposure-response relationship curves between EDVs for urolithiasis and air pollutants and explored the co-effects among six air pollutants.

## 2. Materials and methods

**Figure 1:**
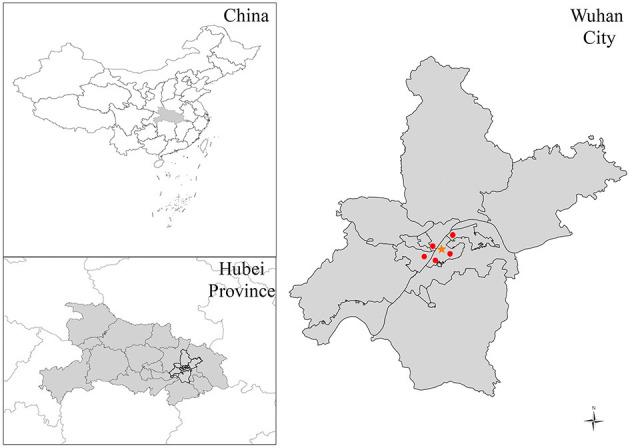
The location of Wuhan, the location of the hospital (the orange star), and monitoring stations (red dots).

Wuhan, the capital of Hubei province, is located in central China (latitude 30°35'N and longitude 114°17'E) and consists of seven central districts and six suburban and rural districts (Figure 1). At the end of 2018, Wuhan had a land area of 8,494.41 square kilometers and a population of 11.1 million (http://www.wuhan.gov.cn). As the core city of the Yangtze River Economic Belt, Wuhan is an essential industrial base and transportation hub in China. Vehicle exhaust and industrial emissions are the primary sources of air pollution. Wuhan has a typical subtropical monsoon climate, with hot and humid summers and cold and dry winters. The average temperature is 4.4°C in January and 30.3°C in July.

### 2.1. Emergency department visits data

The majority of the EDVs of Zhongnan Hospital are from the Wuchang District of Wuhan, where the hospital is located. It is a district that contains 19% of the population of the whole urban area and 12% of the population of Wuhan overall (http://www.wuhan.gov.cn). The daily number of EDVs from January 1, 2016 to December 31, 2018 were obtained from Zhongnan Hospital of Wuhan University in Wuhan, China. The EDV data followed a quasi-Poisson distribution. Outpatient visits diagnosed with urolithiasis (mainly kidney stones) (*N* = 7,483) were selected as our study population. To ensure the accuracy of the study results, we removed duplicate records and non-Wuhan permanent residents and re-matched the tenth version of the International Classification of Diseases, with codes N20-N23. The study protocol was approved by the Medical Ethics Committee of Zhongnan Hospital (IRB number: 20211018K).

### 2.2. Air pollution and meteorological data

Daily ambient air pollution data (SO_2_, NO_2_, PM_2.5_, PM_10_, CO, and O_3_) from January 2016 to December 2018 was obtained from the Wuhan Ecological Environment Bureau website (http://hbj.wuhan.gov.cn/). The daily average concentrations of air pollutants were measured from the fixed-site monitoring stations. Average daily air pollutant concentrations at the monitoring stations were used as a proxy for the overall exposure of all populations. The maximum 8-h average of O_3_ and the daily 24-h average of the remaining pollutants were calculated.

The data of two meteorological parameters (daily average temperature [°C] and relative humidity [%]) during the study period were obtained from the Meteorological Data Sharing Service System of the China Meteorological Administration (Beijing, China). Environmental and meteorological data were missing for 32 days (2.92%). Therefore, dates with missing data were excluded from our study.

### 2.3. Statistical analysis

Generalized additive model (GAM) was used to conduct this time-series study. Because daily emergency visits followed an over-dispersed Poisson distribution, quasi-Poisson regression was used in the GAM. Several covariates were introduced to control for their potential confounding effects. First was a natural cubic smooth function for calendar time, with 7 degrees of freedom (df) per year to exclude long-term and seasonal trends over 2 months (14). Second, natural smooth functions of the two variables were incorporated into the model: mean temperature (6 df) and relative humidity (3 df), to control for the confounding effect of meteorological factors on the correlation between air pollution and urolithiasis EDVs (15). Third, indicators such as public holidays (Holiday) and “day of the week” (*DOW*) were adjusted as dummy variables in the GAM (15).

The model was as follows:


$$\begin{array}{l} \log E(Yt)=\beta Z_{t}+DOW+ns(time,df)+ns(temperature,6)\\ \;+ns(humidity,3)+interecept \end{array}$$

where *E(Yt)* is the estimated daily EDVs for kidney stones at day t; β indicates the log-relative rate of urolithiasis correlated with increased air pollutant units; *Z*_*t*_ represents the pollutant concentrations at day *t*; *DOW* is a dummy variable for the day of the week; *ns* refers to the natural cubic regression smooth function. The exposure-response (E-R) relationship was drawn between air pollutants and urolithiasis EDVs by adding a 3 df natural spline function to the above model.

To test the stability of the model, three sensitivity analyses were performed. First, due to the uncertainty of the optimal lag days, we further introduced a single-day lag, including lag0, lag1, lag2, lag3, lag4, lag5, lag6, and lag7, and a multi-day moving average exposure, including lag0-1, 0-2, 0-3, 0-4, 0-5, 0-6, and 0-7. To determine the optimal lag structure, model fits were calculated based on three statistics: Akaike Information Criterion (AIC), Generalized Cross Validation (GCV), and Partial Autocorrelation Function (PACF). Second, for the smoothness of the temporal trend, we chose alternative df with 4–10 per year. Third, two-pollutant models were built to assess the stability of the effect estimates after adjusting for co-pollutants and added co-pollutants with a correlation coefficient < 0.7 to the two-pollutant model. In addition, three stratified analyses were conducted respectively according to season (warm: April to September; cool: October to March), age (< 45 years, 45–65 years, and ≥65 years), and gender (females, males). The statistical significance of the differences between the strata effect estimates were further tested by calculating 95% confidence intervals as ${\hat{Q}}_{1}-{\hat{Q}}_{2}\pm 1.96\sqrt{{\left(S{Ê}_{1}\right)}^{2}+{\left(S{Ê}_{2}\right)}^{2}}$, where Q_1_ and Q_2_ are the estimates for two categories, and SÊ_1_ and SÊ_2_ are their respective standard errors. All statistical analyses were performed in R software (version 4.1.0) using the MGCV package. Results were two-sided and *p* < 0.05 were statistically significant. The results were expressed as the percentage change in urolithiasis EDVs per 10 μg/m^3^ increase in each pollutant concentrations.

## 3. Result

**Figure 2:**
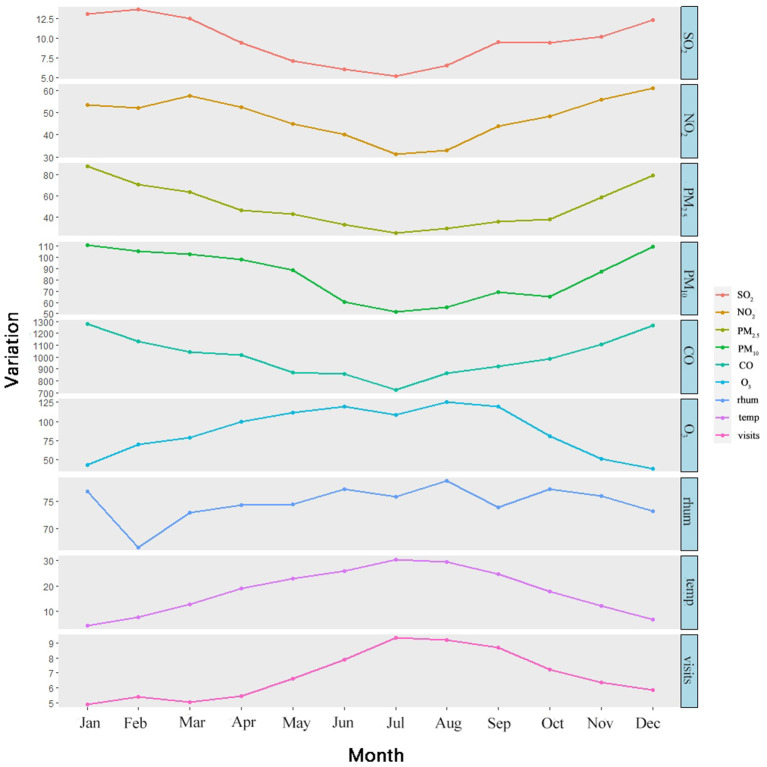
The monthly average variation of pollutants, temperature, relative humidity, and daily emergency department visits for urolithiasis.

Table 1 summarizes the descriptive statistics of EDVs for urolithiasis, air pollutants, and weather data. We collected a total of 7483 cases of urolithiasis admitted to the emergency system of Zhongnan Hospital of Wuhan University from January 1, 2016 to December 31, 2018. The annual mean temperature was 17.87°C and the annual mean humidity was 74.99%. During our study period, an average of seven people presented EDVs for urolithiasis daily. Among all patients, male, female, age < 45 years, age 45–65 years, and age ≥65 years were 72.9, 27.1, 58.6, 33.1, and 8.3% respectively. The EDVs for urolithiasis in warm seasons (57.7%) were higher than those in cool seasons (42.3%). The annual average concentrations of air pollutants were 50.79 μg/m^3^ for PM_2.5_, 83.55 μg/m^3^ for PM_10_, 47.87 μg/m^3^ for NO_2_, 1,005.63 μg/m^3^ for CO, 9.55 μg/m^3^ for SO_2_, and 87.40 μg/m^3^ for O_3_. During our study, there were 229 (20.9%) and 382 (24.8%) days respectively where NO_2_ and PM_2.5_ levels exceeded Chinese secondary ambient air quality standards. Other air pollutants were within the standard range most of the time. The monthly average variation of pollutants, temperature, relative humidity, and daily emergency department visits for urolithiasis are shown in Figure 2.

**Table 1:** The summary of daily air pollutants, weather conditions, and daily emergency department visit for urolithiasis (*N* = 7,483) during our study period (January 1, 2016–December 31, 2018).

	**Mean**	**SD**	**Min**	**P25**	**Median**	**P75**	**Max**
**Air pollutant concentration (**μ**g/m**^3^**)**^a^							
SO_2_	9.55	5.53	2.70	5.50	8.00	12.25	49.50
NO_2_	47.87	19.36	14.00	32.76	45.12	60.96	125.00
PM_2.5_	50.79	30.91	6.93	28.89	43.96	64.47	204.70
PM_10_	83.55	47.49	8.60	48.70	76.50	108.00	561.50
CO	1,005.63	302.68	356	794.00	952.00	1,172.00	2,372.00
O_3_	87.40	47.63	4.20	49.60	80.20	121.41	255.00
**Meteorological measures**							
Temperature (°C)	17.87	9.31	−4.00	10.00	18.89	25.98	35.42
Humidity (%)	74.99	13.38	36.00	65.25	75.75	85.50	100.00
Emergency department visit for kidney stones	7	4	0	4	6	9	25
**Season (** ***N*** **)**							
Warm^b^	8	4	0	4	7	11	25
Cool^c^	6	3	0	3	5	8	24
**Gender (** ***N*** **)**							
Male	5	3	0	2	4	7	19
Female	2	2	0	1	2	3	9
**Age (** ***N*** **)**							
< 45	4	3	0	2	4	6	16
45–65	2	2	0	1	2	3	16
≥65	1	1	0	0	0	1	5

^a^24-h average for PM_2.5_, PM_10_, SO_2_, and NO_2_; maximal 8-h average for O_3_.

^b^Warm season: from April to September.

^c^Cool season: from October to March.

SD, standard deviation; P25, 25th percentile; P75, 75th percentile; PM_2.5_, particulate matter with an aerodynamic diameter less than or equal to 2.5 μm; PM_10_, particulate matter with an aerodynamic diameter less than or equal to 10 μm; O_3_, ozone; SO_2_, sulfur dioxide; NO_2_, nitrogen dioxide; CO, carbon monoxide.

**Figure 3:**
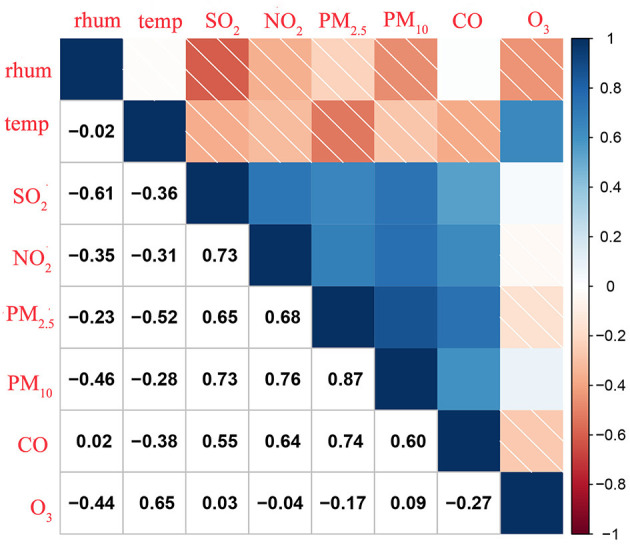
Spearman correlations among exposure variables in Wuhan, China (2016–2018). temp, Temperature; rhum, Relative Humidity.

Generally, there were moderate to strong correlation coefficients among PM_2.5_, PM_10_, SO_2_, NO_2_, and CO (Spearman's correlation coefficient ranged from 0.55 to 0.87), and they all were weakly correlated with O_3_ (−0.27 to 0.09). All pollutants were negatively correlated with temperature except for O_3_ (0.65) and with relative humidity except for CO (0.02) (Figure 3).

**Figure 4:**
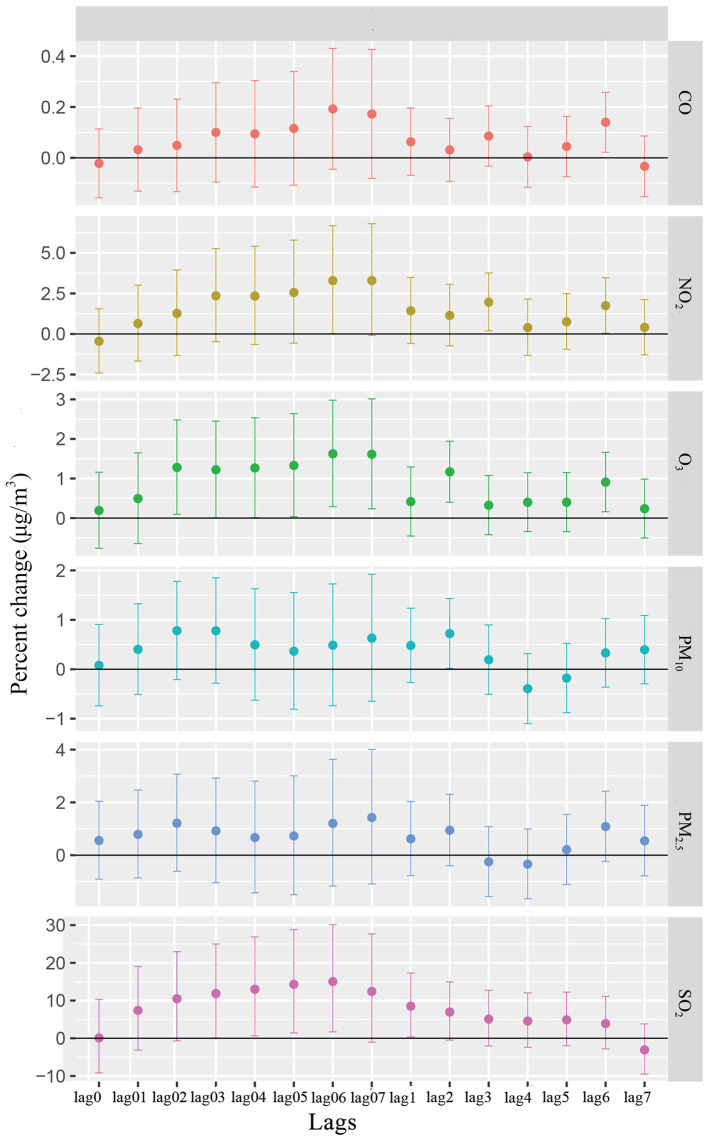
Percentage change (%) of EDVs for urolithiasis (mean and 95%CI) associated with a 10 μg/m^3^ increase in various air pollutant concentrations using different lag structures.

Figure 4 exhibits the percentage change (mean and 95% CI) in urolithiasis EDVs associated with a 10 μg/m^3^ increase in the concentration of six pollutants when using different lag structures—single lag day (lag1-lag7) and moving average lag models (lag01-lag07). Notably, we found statistically significant correlations between emergency hospital admissions for urolithiasis and SO_2_, NO_2_, CO, and O_3_. According to the model fitting statistics, we selected lag06 for SO_2_, lag3 for NO_2_, lag6 for PM_2.5_ and CO, and lag2 for PM_10_ and O_3_ as the best lag structures because they can produce the smallest AIC/GCV/PACF values. A total of 10 μg/m^3^ increases of SO_2_, NO_2_, PM_2.5_, CO, PM_10_, and O_3_ respectively corresponded to increases in daily EDVs for urolithiasis of 15.02% (95% CI: 1.69%, 30.11%), 1.96% (95% CI: 0.19%, 3.76%), 1.09% (95% CI: −0.24%, 2.43%), 0.14% (95% CI: 0.02%, 0.26%), 0.72% (95% CI: 0.02%, 1.43%), and 1.17% (95% CI: 0.40%, 1.94%) (Table 2). The associations of all pollutants with EDVs for urolithiasis were generally positive, but in all lag structures we examined, PM_2.5_ and PM_10_ were statistically insignificant.

**Table 2:** Percent change (95% CI) in urolithiasis EDVs with a 10 μg/m^3^ increase in air pollutant concentrations by season, gender and age in Wuhan, China.

**Pollutants**	**Season**		**Gender**		**Age**		
	**Cool**	**Warm**	**Male**	**Female**	**Younger**	**Middle**	**Elder**
SO${}_{2}^{\text{a}}$	−0.45 (−2.41, 1.55)	40.29 (8.02, 82.21)^*^	12.44 (−2.61, 29.83)	22.43 (−0.54, 50.72)	21.75 (4.50, 41.84)^*^	6.13 (−12.53, 28.77)	0.17 (−32.08, 47.74)
NO${}_{2}^{\text{b}}$	3.84 (1.30, 6.44)^*^	−0.46 (−3.18, 2.33)	1.83 (−0.22, 3.92)	2.31 (−0.71, 5.42)	3.05 (0.83, 5.32)^*^	0.33 (−2.37, 3.10)	0.20 (−5.19, 5.90)
PM${}_{2.5}^{\text{c}}$	0.59 (−1.04, 2.24)	2.08 (−0.59, 4.83)	0.16 (−1.37, 1.70)	3.32 (1.14, 5.54)^*^	1.81 (0.19, 3.45)^**#**^	−0.99 (−3.02, 1.08)^**#**^	3.90 (−0.14, 8.11)^**#**^
PM${}_{10}^{\text{d}}$	0.67 (−0.52, 1.88)^**#**^	0.79 (−0.13, 1.71)^**#**^	0.74 (−0.07, 1.55)	0.61 (−0.58, 1.82)	0.90 (0.04, 1.77)^*^	0.41 (−0.70, 1.52)	0.74 (−0.07, 1.55)
CO ^c^	0.09 (−0.07, 0.24)	0.21 (0.01, 0.41)^*^	0.04 (−0.10, 0.18)	0.34 (0.15, 0.54)^*^	0.15 (0.01, 0.30)^*^	0.00 (−0.18, 0.19)	0.40 (0.04, 0.76)^*^
O${}_{3}^{\text{d}}$	2.47 (0.77, 4.21)^*^	2.47 (0.77, 4.21)^*^	1.30 (0.41, 2.19)	0.81 (−0.53, 2.18) ^*^	1.22 (0.24, 2.21)^*^	1.34 (0.17, 2.52)^*^	0.06 (−2.26, 2.44)

^*^p < 0.05.

^a^Moving average of lag 06 was used for SO_2_.

^b^Single average of lag 3 was used for NO_2_.

^c^Single average of lag 6 was used for PM_2.5_ and CO.

^d^Single average of lag 2 was used for PM_10_ and O_3_.

^**#**^Statistically significant for between group difference.

**Figure 5:**
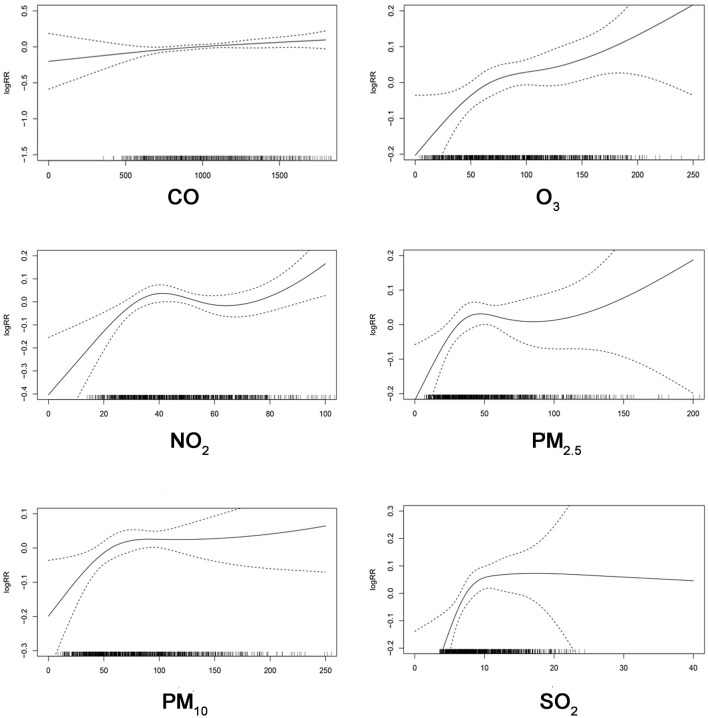
The exposure-response relationship curves of SO_2_, NO_2_, PM_2.5_, PM_10_, CO, and O_3_.

The exposure-response (E-R) curves of air pollutants and urolithiasis EDVs are shown in Figure 5. The E-R relationships of CO and O_3_ were nearly linear, indicating no thresholds for their associations with EDVs for urolithiasis. The curve for NO_2_ showed a steep slope at concentrations < 40 μg/m^3^ and became a J-shape at concentrations > 40μg/m^3^. For the curve of PM_2.5_, a steep slope was observed at concentrations < 50 μg/m^3^, a relatively flat slope at concentrations between 50 μg/m^3^ and 100 μg/m^3^, and became steep again at concentrations > 100 μg/m^3^. The E-R curves of PM_10_ and SO_2_ rose sharply when the concentrations were < 75 μg/m^3^ and < 10 μg/m^3^ and gradually flattened after the concentrations were higher than the critical values.

Table 2 displays how different effect estimates of ambient air pollution on urolithiasis EDVs varied by season, gender, and age. In season-specific analysis, the results suggested that the correlations between urolithiasis EDVs and SO_2_ and CO were generally stronger in warm seasons than in cool seasons, while correlations with NO_2_ were stronger in cool seasons. In gender-specific analysis, stronger correlations between PM_2.5_ and CO and urolithiasis EDVs were observed in females than in males. Among female patients, PM_2.5_, CO, and O_3_ were significantly correlated with urolithiasis EDVs, whereas the results were positive but not significant in male patients. In age-specific analysis, the effects of SO_2_, NO_2_, and PM_10_ tended to be greater for people aged < 45 years, while CO was more apparent in people aged ≥ 65 years, and the correlation of O_3_ was more pronounced in people aged 465 years.

The estimated effects were not significantly affected by adjusting for temporal smoothness using an alternative df from 4 to 10 per year (Supplementary Figure 1). Table 3 shows the correlations between urolithiasis EDVs and air pollution after adding co-pollutants with spearman's correlation coefficients < 0.7 to the two-pollutants model for adjustment. When adjusting for all other pollutants, we found that the risk estimates of CO and O_3_ on urolithiasis EDVs remained statistically significant. The correlations of PM_10_, SO_2_, and urolithiasis EDVs were statistically significant only when controlling for CO. For PM_2.5_, the estimated effects were insignificant regardless of which pollutant was adjusted.

**Table 3:** Percent change (%, mean and 95% CI) in EDVs for urolithiasis in two-pollutant models.

**Two-pollutants**		**Percent change**
SO${}_{2}^{\text{a}}$	–	**15.02 (1.69, 30.11)** ^*^
	+PM_2.5_	13.35 (−0.01, 28.50)
	+CO	**14.33 (1.10, 29.29)** ^*^
	+O_3_	9.51 (-3.72, 24.57)
NO${}_{2}^{\text{b}}$	–	**1.96 (0.19, 3.76)** ^*^
	+PM_2.5_	**1.99 (0.22, 3.79)** ^*^
	+CO	**2.30 (0.51, 4.11)** ^*^
	+O_3_	1.37 (-0.44, 3.22)
PM${}_{2.5}^{\text{c}}$	–	1.09 (-0.24, 2.43)
	+SO_2_	0.83 (-0.52, 2.19)
	+NO_2_	1.11 (-0.21, 2.45)
	+O_3_	1.05 (-0.27, 2.38)
PM${}_{10}^{\text{d}}$	–	0.72 (0.02, 1.43)
	+CO	**0.77 (0.07, 1.48)** ^*^
	+O_3_	−0.30 (−0.36, 1.17)
CO^c^	–	**0.14 (0.02, 0.26)** ^*^
	+SO_2_	**0.13 (0.02, 0.25)** ^*^
	+NO_2_	**0.16 (0.04, 0.28)** ^*^
	+PM_10_	**0.15 (0.03, 0.27)** ^*^
	+O_3_	**0.15 (0.03, 0.26)** ^*^
O${}_{3}^{\text{d}}$	–	**1.17 (0.40, 1.94)** ^*^
	+SO_2_	**0.99 (0.19, 1.81)** ^*^
	+NO_2_	**1.02 (0.23, 1.82)** ^*^
	+PM_2.5_	**1.16 (0.39, 1.93)** ^*^
	+PM_10_	**1.02 (0.20, 1.84)** ^*^
	+CO	**1.20 (0.43, 1.97)** ^*^

^*^p < 0.05.

^a^Moving average of lag 06 was used for SO_2._

^b^Single average of lag 3 was used for NO_2._

^c^Single average of lag 6 was used for PM_2.5_ and CO.

^d^Single average of lag 2 was used for PM_10_ and O_3._

## 4. Discussion

According to statistics, the prevalence of urolithiasis is gradually rising (16). Due to the acute nature of presentation, urolithiasis generates a large number of emergency department visits and hospital admissions (17). In recent years, numerous epidemiological studies have reported the positive correlations between air pollution and the development of urological diseases. However, few studies have explored the correlations between air pollutants and urolithiasis. Our research demonstrated a significant correlation between short-term exposure to air pollutants, including SO_2_, NO_2_, CO, and O_3_ and increased risks of urolithiasis. The correlations between CO, O_3_ and urolithiasis EDVs were robust with co-pollutants adjustment. The correlations were found mainly in females (especially from PM_2.5_ and CO) and in younger people (especially from SO_2_, NO_2_, and PM_10_). In contrast, the correlation between O_3_ and urolithiasis EDVs was more pronounced in males and middle-aged people, and the correlation of CO was more evident in elders. The effects of SO_2_ and CO were stronger in warm seasons than in cool seasons, while the effect of NO_2_ was more substantial in cool seasons. Our findings have added to the limited evidence that air pollution may be a potential risk factors for the incidence of urolithiasis.

During our study period, the annual average concentrations of NO_2_ (58.89 μg/m^3^) and PM_2.5_ (47.87 μg/m^3^), and PM_10_ (83.55 μg/m^3^) in Wuhan exceeded China's National Ambient Air Quality standards (40 μg/m^3^, 40 μg/m^3^ and 15 μg/m^3^). Since Wuhan is the largest city in central China, the expansion of cities, the development of industry, the increase of motor vehicles, and the growth of population may all be contributors to the severe air pollution.

Our study observed positive correlations between air pollutants (including SO_2_, NO_2_, PM_2.5_, PM_10_, CO, and O_3_) and the EDVs for urolithiasis, especially SO_2_, NO_2_, CO and O_3_, which is partially consistent with previous studies. Oxidative stress may be a primary mechanism of the positive correlations (18). Studies have shown that air pollutants, including O_3_, SO_2_, CO, NO_2_, and PM, are sources of several ROS and other byproducts of oxidative stress (19). In kidneys, they can cause damage to renal tubular epithelial cells and participate in the modification of crystalloid regulators (13, 20, 21), further affecting the formation of calcium oxalate crystals and urinary stones (22–25). Another potential mechanism is that air pollutants can affect urinary tract metabolites (26, 38), and specific urinary tract metabolites such as dimethyl-L-arginine and 2-oxo-arginine are thought to be associated with the formation of kidney stones (27). In addition, immune damage caused by gaseous pollutants may also play an important role (28). Macrophage-associated immune damage is a major change in the immune response observed in renal stone disease (2) and may promote the formation of Randall's plaque (calcified spots appearing in the renal papillae before the formation of kidney stones) and calcium oxalate stones (29). However, the exact biological mechanism remains unknown and warrants further study.

Our findings are partially consistent with those of previous studies. For example, a time series study conducted in Korea mentioned that PM_2.5_ and CO might be new potential risk factors for urolithiasis (18). A nationwide time-series study in China observed consistent associations between same-day PM_2.5_ and several genitourinary diseases, including nephritis, nephrosis, and renal sclerosis; chronic renal failure; and calculus of urinary tract (30). However, PM_2.5_ did not demonstrate a significant correlation with urolithiasis in our study, which may due to different study designs, study populations, confounding factors, such as outdoor activities and dietary habits on disease, and the characteristics of different urban air pollution mixtures (31).

Our study further demonstrated the association between short-term exposure to air pollutants and daily EDVs for urolithiasis varied according to season, age and gender. It is observed that the effects of SO_2_ and CO on urolithiasis EDVs were stronger in warmer seasons. This may be attributed to the physiological impact of temperature itself. Mechanisms of this correlation include insufficient fluid intake, increased sweating, and water evaporation, which lead to concentrated urine and further promote calcium, oxalic acid, uric acid, phosphate crystallization, and stone formation (32, 33). It is supposed the difference that NO_2_ showing higher effects in cool seasons might be related to varying concentrations, sources and composition of air pollution in different seasons (34).

In age stratification, our study indicates that SO_2_, NO_2_ and PM_10_ tended to have more significant effects in people aged < 45 years, which is contrary to the epidemiological evidence of a high incidence rate of urolithiasis in the elderly (35). This may be because young people usually have more outdoor activities, increasing their exposure to air pollutants. However, O_3_ and CO would significantly increase the risk of urolithiasis in middle-aged and older adults. In gender stratification, it is found that females are more vulnerable to PM_2.5_ and CO, while males are more susceptible to O_3_. We cannot find sufficient evidence in the literature for the phenomenon that different individual pollutants have a maximum impact on people of different ages and genders. Since individual pollutants are rarely produced in isolation, so sorting out the effects of individual pollutants is severely limited by potential confounding factors. Observational studies based on populations should consider correlations between them (36).

Interpreting trends in exposure-response relationships is critical to assessing public health. In the present research, threshold concentrations were not observed above which NO_2_ and PM_2.5_ were not correlated with urolithiasis EDVs. It is worth noting that the E-R curve for SO_2_ flattens out at high concentrations. This may result from a “harvest effect”, where sensitive populations might have already been affected and gone to the hospital before air pollutant concentrations reached significant levels (37). Multiple factors such as location, population sensitivity, air pollution mixtures, and climate characteristics may impact the E-R relationship (31). Therefore, more research is still needed in the future to explore the characteristics of the E-R relationship.

Our study has some noted limitations. First, average concentrations of air pollutants measured by stationary site monitoring were used to represent individual exposures, leading to misclassification of exposures and ignoring the spatial effect of air pollution on urolithiasis. Secondly, some factors were not taken into account that may affect the formation of urolithiasis and impair a person's tolerance to air pollutants, such as dietary habits, lifestyle, and metabolic syndrome. As a result, combined estimates may skew the effects of air pollutants in specific populations. Third, the data we collected was only from a highly polluted city; thus, the general study application of study may be limited. Therefore, further studies are needed to confirm our results, and molecular biology or animal experiments are necessary to explore the exact mechanisms between air pollutants and urinary stones formation.

## 5. Conclusion

Our time-series study found that ambient air pollution (especially SO_2_, NO_2_, CO, and O_3_) positively correlated with the EDVs for urolithiasis in Wuhan, China. These effects were found to vary by season, gender, and age. Our study adds limited evidence to how air pollutants affect urolithiasis and we hope this study can support hospitals and clinical staff in taking effective preventive measures to maintain emergency department order, particularly on heavily polluted days.

## Data availability statement

The original contributions presented in the study are included in the article/Supplementary material, further inquiries can be directed to the corresponding author.

## Ethics statement

The studies involving human participants were reviewed and approved by the Medical Ethics Committee of Zhongnan Hospital (IRB number: 20211018K). Written informed consent for participation was not required for this study in accordance with the national legislation and the institutional requirements.

## Author contributions

HX: methodology, software, visualization, and writing—original draft. YL: investigation, writing—reviewing, and editing. JW: illustration production. XJ: supervision, project administration, and funding acquisition. All authors commented on previous versions of the manuscript and read and approved the final manuscript.

## Supplementary material

Supplementary Figure 1 Percentage change (%) of EDVs for urolithiasis (mean and 95%CI) associated with a 10 μg/m^3^ increase in various air pollutant concentrations at their peak lag day using different degrees of freedom per day.

Supplementary Table 1 Percent change (mean and 95% CI) in EDVs for urolithiasis associated with a 10 μg/m^3^ increase in concentrations of six pollutants using different lag structures.
