# Supplementary figures and images for: Short-term effects of ambient air pollution on emergency department visits for urolithiasis: A time-series study in Wuhan, China
Source: Front Public Health. 2023 Jan 30;11:1091672. doi: 10.3389/fpubh.2023.1091672 (PMC9922887; doi:10.3389/fpubh.2023.1091672)

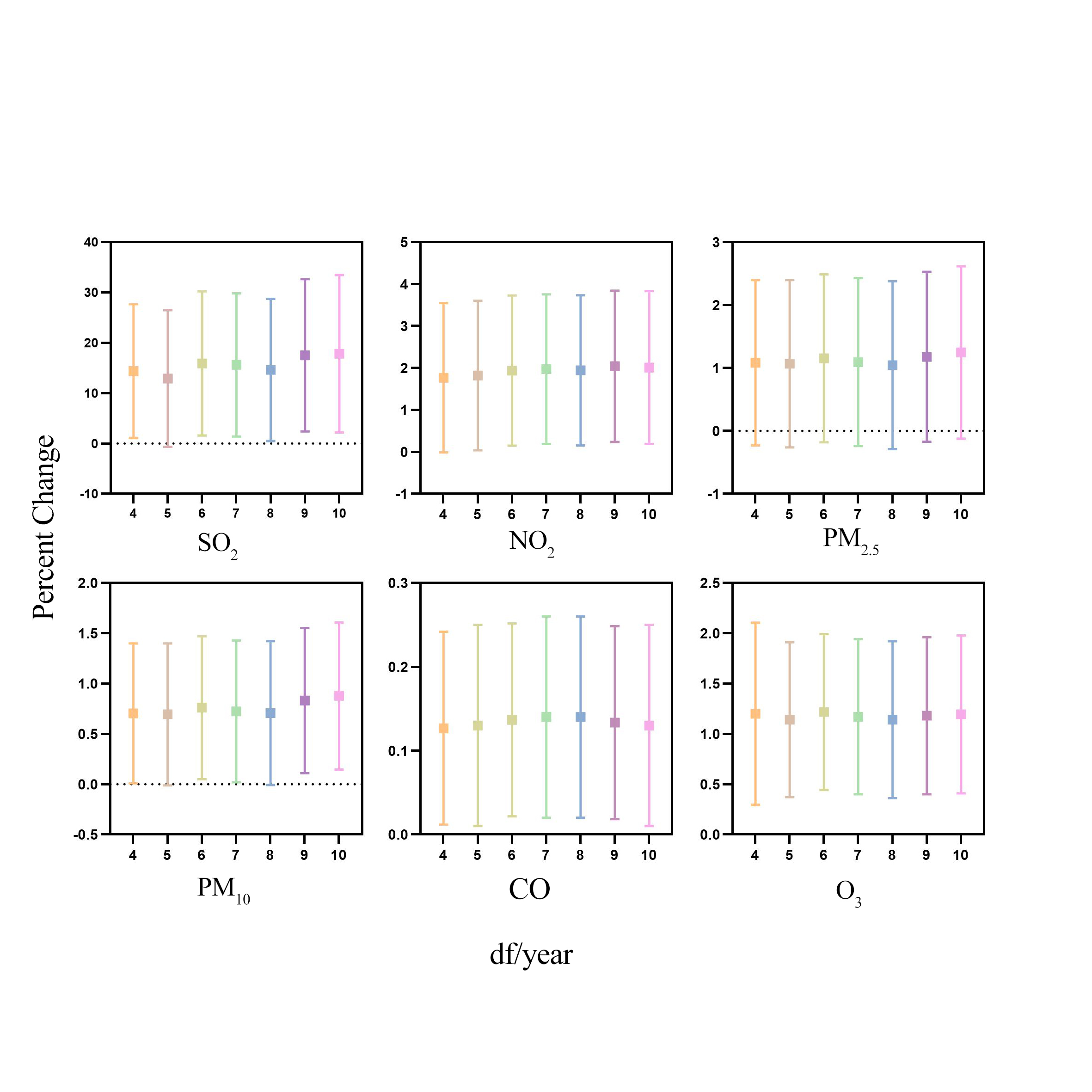

Supplement: Supplementary Figure 1 — Percentage change (%) of EDVs for urolithiasis (mean and 95%CI) associated with a 10 μg/m3 increase in various air pollutant concentrations at their peak lag day using different degrees of freedom per day. [file Image_1.JPEG]
