# Supplementary material for: Short-term effects of ambient air pollution on emergency department visits for urolithiasis: A time-series study in Wuhan, China
Source: Front Public Health. 2023 Jan 30;11:1091672. doi: 10.3389/fpubh.2023.1091672 (PMC9922887; doi:10.3389/fpubh.2023.1091672)
Supplement: Supplementary Table 1 — Percent change (mean and 95% CI) in EDVs for urolithiasis associated with a 10 μg/m3 increase in concentrations of six pollutants using different lag structures. [file Table_1.DOCX]

**Table S1** Percent change (mean and 95% CI) in EDVs for urolithiasis associated with a 10-μg/m^3^ increase in concentrations of six pollutants using different lag structures.

| Lag | SO_2_ | NO_2_ | PM_2.5_ | PM_10_ | CO | O_3_ |
| --- | --- | --- | --- | --- | --- | --- |
| 0 | 0.11 (-9.16,10.32) | -0.45 (-2.41,1.55) | 0.56 (-0.91,2.04) | 0.08 (-0.74,0.91) | -0.02 (-0.16,0.11) | 0.19 (-0.76,1.16) |
| 1 | **8.51 (0.38,17.29)*** | 1.43 (-0.58,3.49) | 0.62 (-0.77,2.03) | 0.48 (-0.27,1.24) | 0.06 (-0.07,0.20) | 0.42 (-0.45,1.29) |
| 2 | 6.95 (-0.49,14.96) | 1.14 (-0.74,3.06) | 0.95 (-0.40,2.31) | 0.72 (0.02,1.43) | 0.03 (-0.09,0.15) | **1.17 (0.40,1.94)*** |
| 3 | 5.08 (-2.04,12.72) | **1.96 (0.19,3.76)*** | -0.25 (-1.57,1.08) | 0.19 (-0.51,0.90) | 0.09 (-0.03,0.20) | 0.32 (-0.42,1.08) |
| 4 | 4.57 (-2.39,12.03) | 0.40 (-1.33,2.15) | -0.34 (-1.65,0.99) | -0.39 (-1.10,0.32) | 0.00 (-0.12,0.12) | 0.40 (-0.34,1.15) |
| 5 | 4.88 (-1.99,12.24) | 0.75 (-0.95,2.49) | 0.21 (-1.11,1.55) | -0.18 (-0.88,0.52) | 0.04 (-0.07,0.16) | 0.40 (-0.34,1.15) |
| 6 | 3.91 (-2.82,11.11) | **1.75 (0.05,3.47)*** | 1.09 (-0.24,2.43) | 0.33 (-0.36,1.03) | **0.14 (0.02,0.26)*** | **0.91 (0.16,1.66)*** |
| 7 | -3.06 (-9.49,3.82) | 0.40 (-1.29,2.12) | 0.54 (-0.78,1.89) | 0.39 (-0.30,1.09) | -0.03 (-0.15,0.09) | 0.24 (-0.50,0.99) |
| 01^a^ | 7.39 (-3.12,19.04) | 0.65 (-1.67,3.02) | 0.79 (-0.86,2.47) | 0.40 (-0.51,1.32) | 0.03 (-0.13,0.20) | 0.49 (-0.65,1.65) |
| 02 ^a^ | 10.49 (-0.69,22.93) | 1.27 (-1.33,3.95) | 1.21 (-0.61,3.07) | 0.78 (-0.21,1.78) | 0.05 (-0.13,0.23) | **1.28 (0.09,2.48)*** |
| 03 ^a^ | **11.85 (0.09,25.00)*** | 2.36 (-0.47,5.26) | 0.92 (-1.04,2.92) | 0.78 (-0.28,1.85) | 0.10 (-0.10,0.30) | **1.22 (0.01,2.45)*** |
| 04 ^a^ | **13.00 (0.66,26.86)*** | 2.34 (-0.65,5.41) | 0.67 (-1.43,2.81) | 0.49 (-0.63,1.63) | 0.09 (-0.12,0.30) | **1.27 (0.01,2.54)*** |
| 05 ^a^ | **14.31 (1.41,28.84)*** | 2.56 (-0.57,5.79) | 0.73 (-1.50,3.01) | 0.36 (-0.81,1.55) | 0.12 (-0.11,0.34) | **1.33 (0.03,2.64)*** |
| 06 ^a^ | **15.02 (1.69,30.11)*** | **3.29 (0.02,6.67)*** | 1.20 (-1.17,3.63) | 0.49 (-0.74,1.73) | 0.19 (-0.05,0.43) | **1.63 (0.29,2.98)*** |
| 07 ^a^ | 12.41 (-1.02,27.67) | 3.30 (-0.09,6.80) | 1.43 (-1.09,4.01) | 0.63 (-0.65,1.92) | 0.17 (-0.08,0.43) | **1.61(0.23,3.01)*** |

***Note.*** The statistically significant estimates are highlighted in bold.

^a^ Lag 01(02,03,04,05,06,07) the moving average concentrations on the present day and previous 1(2,3,4,5,6,7) day.
